# Stability of Willingness to Pay: Does Time and Treatment Allocation in a Randomized Controlled Trial Influence Willingness to Pay?

**DOI:** 10.1177/0272989X241249654

**Published:** 2024-05-13

**Authors:** Marjon van der Pol, Verity Watson, Dwayne Boyers

**Affiliations:** Health Economics Research Unit, University of Aberdeen, Aberdeen, UK (MVDP, VW, DB); Health Economics Research Unit, University of Aberdeen, Aberdeen, UK (MVDP, VW, DB); Health Economics Research Unit, University of Aberdeen, Aberdeen, UK (MVDP, VW, DB)

**Keywords:** contingent valuation, dentistry, stability, willingness to pay

## Abstract

**Background:**

Willingness-to-pay (WTP) estimates are useful to policy makers only if they are generalizable beyond the moment when they are collected. To understand the “shelf life” of preference estimates, preference stability needs be tested over substantial periods of time.

**Methods:**

We tested the stability of WTP for preventative dental care (scale and polish) using a payment-card contingent valuation question administered to 909 randomized controlled trial participants at 4 time points: baseline (prerandomization) and at annual intervals for 3 years. Trial participants were regular attenders at National Health Service dental practices. Participants were randomly offered different frequencies (intensities) of scale polish (no scale and polish, 1 scale and polish per year, 2 scale and polishes per year). We also examined whether treatment allocation to these different treatment intensities influenced the stability of WTP. Interval regression methods were used to test for changes in WTP over time while controlling for changes in 2 determinants of WTP. Individual-level changes were also examined as well as the WTP function over time.

**Results:**

We found that at the aggregate level, mean WTP values were stable over time. The results were similar by trial arm. Individuals allocated to the arm with the highest scale and polish intensity (2 per year) had a slight increase in WTP toward the latter part of the trial. There was considerable variation at the individual level. The WTP function was stable over time.

**Conclusions:**

The payment-card contingent valuation method can produce stable WTP values in health over time. Future research should explore the generalizability of these results in other populations, for less familiar health care services, and using alternative elicitation methods.

**Highlights:**

- Stated preferences are commonly used to value health care.
- Willingness-to-pay (WTP) estimates are useful only if they have a “shelf life.”
- Little is known about the stability of WTP for health care.
- We test the stability of WTP for dental care over 3 y.
- Our results show that the contingent valuation method can produce stable WTP values.

The absence of well-functioning markets in health care means that there are very limited opportunities to measure revealed preferences through observing behavior. This is similar to other areas with nonmarket goods such as the environment. Health economists therefore apply survey-based, stated preference methods to value health and health care.^1,2^ Stated preference methods assume that individuals’ responses to hypothetical valuation tasks are based on complete, stable, and rational preferences that are consistent with the axioms of utility theory. Preference estimates are useful to policy makers only if they are generalizable beyond the moment when they are collected. Tests of preference stability over substantial periods of time are needed to understand the “shelf life” of preference estimates.^3^ However, most previous contingent valuation (CV) studies of test-retest reliability in health care have short time periods of less than 6 wk.^4–8^ Two exceptions are Thompson et al.^9^ and Settumba et al.,^10^ who tested reliability over 12 and 10 mo, respectively. Two studies explored the stability of willingness to pay (WTP) in discrete choice experiment tasks.^11,12^ Skjoldborg et al.^11^ found no significant differences in marginal WTP for attributes of rheumatoid arthritis treatment elicited at 3 time points up to 4 mo apart. Price et al.^12^ found no differences in marginal WTP for attributes describing the mortality and morbidity reduction from improved tap water quality elicited from different samples at 3 time points 8 y apart. Preference stability tests of WTP conducted over longer time periods are needed to understand the shelf life of preference estimates.

WTP is expected to change under certain circumstances.^13^ WTP elicited in response to the same stated preference tasks across time points should be unchanged if the determinants of WTP (such as income, price of complements and substitutes, inflation, etc.) are unchanged. However, WTP is expected to change if the determinants of preferences change. For example, a large reduction in income should reduce an individual’s WTP. WTP is also expected to change over time if the relationship between determinants and WTP change.^3^ While a short duration between valuation surveys reduces the likelihood that determinants or the relationship between determinants and WTP change between waves, such changes are still possible.^7,14^ Any unexpected changes in WTP values in health care may suggest that the method itself is not able to elicit robust stable values in health care. However, unexpected changes in WTP values may also occur if individuals are unfamiliar with the good and have incomplete preferences. Individuals may become more familiar with the good over time, and this can influence the stability of their WTP values. To better understand whether the elicitation method itself can produce stable estimates in health care, we examine the stability of WTP for a familiar health care good (scale and polish). We do this in a sample of regular attenders at UK National Health Service (NHS) dental practices who have experience with the good that is being valued.

We use a unique data set in which WTP was elicited at 4 time points over a relatively long period (baseline and at annual intervals for 3 years). We compare the average WTP as well as the WTP function over time. The data were collected as part of a randomized controlled trial (RCT) that also allows us to examine whether context matters. Participants were randomly offered different frequencies (intensities) of scale polish (no scale and polish, 1 scale and polish per year, 2 scale and polishes per year). Being allocated to different treatment intensities should not affect WTP if individuals’ responses to hypothetical valuation tasks are based on complete, stable, and rational preferences that are consistent with the axioms of utility theory. However, it can be hypothesized that WTP may be affected in at least 2 ways. First, being allocated to the no scale and polish arm may lead to disappointment and what has been termed *resentful demoralization*.^15,16^ Trial participants who do not receive their preferred treatment allocation may be less motivated and may not report accurately during follow-up. This may lead to instability in WTP values. Their reported WTP before allocation to a treatment arm may therefore be different from their reported WTP after allocation. Second, the differences in intensity across arms can lead to differences in experience, and this may have an impact on stated WTP. Utility theory assumes that individuals make decisions with full information. Unlike choices about daily essentials such as groceries, individuals seldom make decisions about health care goods and services. In this case, individuals may not have complete preferences for these unfamiliar goods and services.^17^ As individuals gain experience of the good or service, they may learn about their preferences.^18–21^ Information and (familial) experience of the health condition has been shown to influence WTP.^22–24^ However, as a scale and polish is a familiar good and given that the study was part of a pragmatic trial (scale and polish was not withheld from patients requesting it, and patients could also obtain additional private scale and polish treatments). we hypothesize that any differences in WTP are more likely to be caused by the allocation itself rather than differences in the frequency of service experienced.

The aim of this article is to test the stability of WTP values over time in a familiar health care good over a long time period and whether treatment allocation to different treatment intensities (0, 1, or 2 treatments per year) influences WTP.

## Methods

### The iQuaD Trial

The data are from the Improving the Quality of Dentistry (IQuaD) multicenter pragmatic split-plot randomized open trial with a cluster factorial design.^25^ Sixty-three NHS dental practices across Scotland and northeast England were randomized to provide routine or personalized oral hygiene advice. Within these dental practices, participants were randomized to 3 groups that were offered different frequencies of NHS provided scale and polish (none, 1 per year, or 2 per year for 3 y). A scale and polish is the thorough cleaning of teeth and gums by a dentist or dental hygienist. Scaling removes hard tartar from teeth, and polishing helps to clean stains off tooth surfaces. It is one of the most frequently provided dental procedures in the United Kingdom. In England, in 2019–2020, 45% of all adult courses of treatment delivered in primary care included a scale and polish as part of the treatment course.^26^ In line with usual practice, participants were required to contribute to the cost of their NHS dental care, unless they were exempt from paying charges. The treatments were provided by NHS dentists and hygienists.

### Sample

The participants were dentate adults who were regular NHS attenders (attended for a dental checkup in the previous 2 y) and who did not have severe gum disease. In total, 1,877 trial participants were recruited. Dental practices sent out invitation letters, a patient information sheet, and a baseline questionnaire (including the CV task) to potentially eligible participants. The study team obtained consent from potentially eligible participants and then collected the baseline clinical measurements and questionnaires. The baseline measurements took place between February 2012 and July 3013. All trial participants received a scale and polish at baseline, after completing the baseline questionnaire (including CV task) and before trial allocation was known. A letter was sent to all participants to inform them of their scale and polish allocation.

Participants completed a questionnaire at baseline (prerandomization) and at annual intervals for 3 y of follow-up. All questionnaires were self-completed postal questionnaires. Of the 1,873^i^ trial participants, 1,119 (59.7%) returned the self-complete questionnaires at all time points. Most of these (81.2%) completed the CV question in each year (*N* = 909). This means that complete CV data are available for 48.5% of trial participants.

### The CV Task

A payment card CV task was used to elicit each participant’s WTP for scale and polish. The payment card method is commonly used to elicit WTP for health care.^23^

The good or service was first described to respondents. A scale and polish is a familiar service for regular dental attenders. The following information was provided in the information sheet: “It is well known that dental plaque is the main cause of gum disease. Effective oral hygiene (tooth brushing and inter-dental aids) for plaque control and the removal of calculus (tartar) by your dentist or hygienist with a scale and polish are considered necessary to prevent and treat gum disease.”

**Figure 1:**
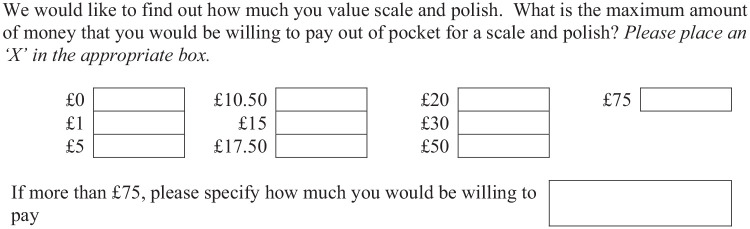
Payment card contingent valuation question included in all waves.

The CV question presented to respondents is shown in Figure 1. The same question was used at all time points. The bid levels in the payment card were chosen as follows. A lower bound of £0 was included to allow that respondents may not value the service. The upper bound of £75 was selected based on the maximum private price for scale and polish treatment across UK providers on an internet price comparison Web site.^27^ The remaining bids were selected to cover the range using an exponential scale.^28^ The bids were then rounded to the nearest whole-pound multiple of £5. The bids £10.50 and £17.50 were added as these were the average patient co-charge in Scotland and England at the time of the study design (2012), rounded to the nearest 50p, respectively.

### Analysis

We tested for stability of WTP over time and the WTP function over time using regression analysis.

#### Regression method

The payment card response data provide an interval-censored signal about the WTP for a scale and polish of individual *i* in time period *t*.^29^ We assume that respondents’ WTP (*WTP_it_*) falls in the interval $b_{\mathrm{it},l}\leq \mathrm{WT}P_{\mathrm{it}}<b_{\mathrm{it},u}$ where *b_it,l_* is the highest bid amount for which respondent *i* places an X in the box and *b_it,u_* is the next highest bid level. The exact WTP value is not known, but the interval within which the value lies is observed. Interval regression fits a linear model to interval-censored data such as the WTP data collected in this study. The coefficients from interval regression can be interpreted the same as in ordinary least squares. INTREG in Stata 15.0 was used to estimate the models.

#### Comparing WTP over time

To examine the stability of WTP over time relative to the baseline, we included 3 dummy variables (year 1, year 2, year 3) in the interval regression model. Statistically significant coefficients on 1 of more of the dummy variables indicate the instability of WTP values. We also conducted a Wald test for joint significance of all 3 dummy variables. Dummy variables may be jointly significant even if they are not individually statistically significant.

We would expect individuals’ WTP to change over time if they experience a change in characteristics that affect WTP. It was therefore important to include time-varying characteristics that are hypothesized to influence WTP. We included 2 time-varying characteristics. First, we included whether a respondent is exempt from co-charges.^ii^ We defined this as a dummy variable that takes the value of 1 if a respondent is exempt and zero otherwise. Exemption was associated with respondents’ socioeconomic status.^iii^ Information on income was not available as the data were from participant trial questionnaires, which generally do not collect this type of information. However, a change in exemption status is likely to represent a large income shock, which would be expected to change an individual’s WTP. It was hypothesized that those who are exempt would have a lower WTP. People who are exempt may also be less familiar with paying for dental care, which may have an impact on their WTP. Second, we included whether the individual uses an electric toothbrush (dummy variable: electric toothbrush) or not. It was hypothesized that individuals who are willing to buy an electric toothbrush care more about their dental health than those who are not and therefore would have higher WTP.

We used fixed-effects interval regression as this allowed us to test the stability of WTP while controlling for respondents’ characteristics that are constant over time:

$$\mathrm{WT}P_{i}=f\left(X_{\mathrm{it}},\;t\right)+i\alpha_{i}+\varepsilon_{\mathrm{it}}$$

where $$( {X_{it}})$ are the time-varying characteristics, individual fixed effect $\left(i\alpha_{i}\right)$ is the individual fixed effect, and $\varepsilon_{\mathrm{it}}$ is an error term. We repeated the analysis by RCT arm to test whether stability over time varied across the RCT arms. The analysis presented used a balanced panel of respondents who completed the payment card task at all 4 time points.

#### Hypotheses

During the data collection period (2012 to 2016), inflation was low (around 1.4%) and there were no major macroeconomic or oral health information shocks. It was therefore hypothesized that the average WTP would be stable over this period. We hypothesized that being allocated to the lowest treatment intensities would be associated with the largest change in WTP, especially at year 1 due to resentful demoralization.

#### Comparing WTP function over time

An individuals’ WTP may not be stable if the relationship between individual characteristics and WTP changes over time. For example, new information may become available that scale and polish is particularly important for older individuals. This means that the relationship between age and WTP for scale and polish may change and, as a result, mean WTP may change over time. We estimated a separate WTP function for each time point. In each case, we estimated an interval regression model as in section Comparing WTP over time, except without fixed effects. We included exemption status and the use of an electric toothbrush as well as several baseline covariates, namely, age (dummy variables: age 35–44 y, age 45–54 y, age 55–64 y, age > 65 y; omitted category age 17–34 y), gender (dummy variable: male), whether the practice employs a dental hygienist or not (dummy variable: dental hygienist), and UK country (dummy variable: England). Previous evidence suggests that some individuals base their WTP responses on the estimated cost of the service (see, for example, Donaldson et al.^30^). Country was therefore included as co-charges vary across England and Scotland. Patient co-charges are higher in England (if the patient has, for example, a checkup and scale and polish only) compared with Scotland (where there are no co-charges for checkups), and it was therefore hypothesized that WTP may be higher in England if responses are influenced by actual service cost to participants. A Chow test was used to test whether the coefficients in the baseline WTP function were statistically significantly different from the coefficients at each of the 3 later time points (year 1, year 2, and year 3).

#### Individual-level changes

The main motivation for this article was to test the “shelf life” of the values at the population average to inform cost-benefit analyses. However, demonstrating the stability of WTP over time at the mean level does not exclude the possibility of changes in WTP at the individual level that are cancled out at the mean level. To explore individual-level changes, we report the number of respondents with no change in bid amount chosen compared with baseline, a 1-interval increase in bid amount chosen (for example, from £10.50 to £15), 2-or-more-interval increase in bid amount chosen, 1-interval decrease in bid amount chosen, and 2-or-more-interval decrease in bid amount chosen. It could be argued that smaller changes are more likely to be due to imprecision in preferences whereas larger changes may be more likely to indicate a change in WTP. We also report the difference between the maximum and minimum bid amount chosen across all years.

#### Robustness checks

We performed robustness checks of the main analysis. First, we excluded respondents who reported zero WTP from the analysis. Those respondents who reported a WTP of £0 may be protest respondent.^31^ Due to space constraints within the trial questionnaires, we were unable to include any follow-up questions to the CV tasks to understand whether any £0 responses were protests. We therefore rerun the analysis, removing all £0 responses. Second, the analysis was estimated using an unbalanced panel. This can provide an indication as to whether there is a selection bias due to nonresponse.

## Results

Table 1 shows the baseline characteristics of the sample of participants who completed all WTP questions. Most of the sample (68.4%) preferred to have 2 or more scale and polishes per year at their stated maximum WTP at baseline. Of the sample, 92.4% visited their NHS dentist in the past year, and 61.3% had a scale and polish at their last visit. There were more females than males in the sample, and most of the sample was resident in Scotland. Appendix 1 shows the baseline characteristics of the total sample and those respondents who had 1 or more missing WTP responses. Note that missing values on the WTP questions were mainly due to respondents not returning the full trial questionnaire and were therefore not directly related to the WTP question (see the Methods section).

**Table 1.** Characteristics of Participants (Complete Cases *N* = 909)

	*n*	%
Age, y		
<35	113	12.4
35–44	133	14.6
45–54	217	23.9
55–64	239	26.3
≥65	207	22.8
Gender		
Female	586	64.5
Male	323	35.5
Exempt from dental charges		
Nonexempt	774	85.1
Exempt	135	14.9
Uses electric brush		
No	584	64.2
Yes	311	34.2
Missing	14	1.5
Practice employs a hygienist		
No	214	23.5
Yes	695	76.5
Country		
Scotland	642	70.6
England	267	29.4
Date of last visit to dentist		
<1 y ago	840	92.4
1–2 y ago	60	6.6
>2 y ago	5	0.6
Missing	4	0.4
How often prefer to have scale and polish		
More than 2 a year	192	21.1
2 a year	430	47.3
Once a year	208	22.9
Once every 2 y	32	3.5
Never	19	2.1
Missing	28	3.1
Scale and polish at last visit		
Yes	557	61.3
No	339	36.3
Missing	22	2.4

Table 2 shows the frequencies of the CV responses at baseline, and Appendix 2 shows the frequencies of the CV responses across all time points. There were relatively few zero responses. Only 6 respondents reported a WTP of £0 at all time points. All individuals who ticked £0 then indicated that they would like to receive a scale and polish. None of the individuals in our balanced panel reported that they were willing to pay more than £75. There is approximately a normal distribution in terms of distribution of responses by bid amount apart from the gap at £17.50. This may the result of the prominence effect, in which respondents are more likely to choose prominent numbers such as 1, 2, 5, 10, 20, and 50.^32^

**Table 2:** Contingent Valuation Task Responses at Baseline (*N* = 909)

	Total	
Bid Amount, £	*n*	%
0	21	2.3
1	1	0.1
5	71	7.8
10.5	181	19.9
15	202	22.2
17.5	55	6.1
20	255	28.1
30	102	11.2
50	19	2.1
75	2	0.2

**Figure 2:**
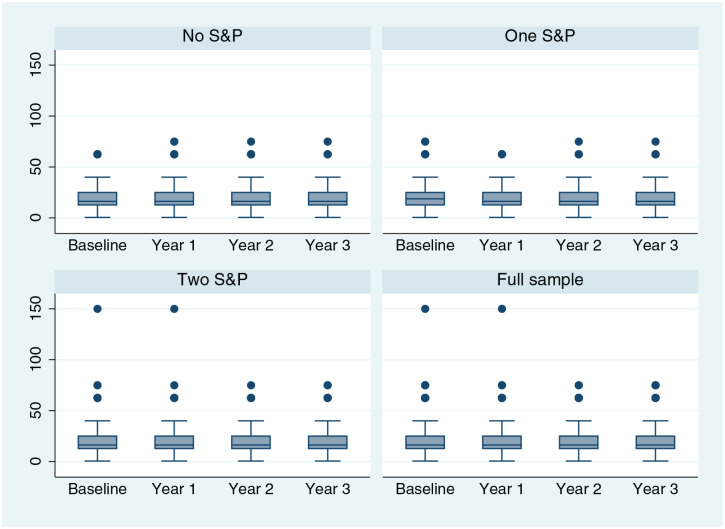
Boxplots of willingness to pay (midpoint) by year and arm.

Figure 2 shows the boxplots of mean WTP by year and randomized allocation (assuming the midpoint of the interval), and Appendix 3 shows the descriptive statistics of mean WTP by year and arm. The mean WTP seemed similar across time points and across arms with confidence intervals clearly overlapping, suggesting that WTP is relatively stable. However, these summary statistics do not control for changes in circumstances over time.

### Comparing WTP over Time

Table 3 shows the results of the fixed-effects interval regression model for the balanced panel (Appendix 4 shows the full regression results). The first model included the full sample and includes the time dummies and covariates. The time dummies were not statistically significant, indicating that mean WTP was stable over time for the whole sample. The model was then estimated by RCT arm to examine whether the stability of WTP varied across trial arms. WTP was relatively stable over time in all trial arms, suggesting that allocating to different treatment intensities did not have a significant impact on WTP. Only 3 of the 9 coefficients were statistically significant at the 5% level. WTP was £1.36 lower at the end of year 1 compared with baseline in the 1 scale and polish arm. Given a mean WTP of £20.53 at baseline, this means that WTP was 6.6% lower. WTP was £0.94 higher at the end of year 2 and £1.09 higher at the end of year 3 compared with baseline in the 2 scale and polish arm. Given a mean WTP of £18.96 at baseline, this means that WTP was 5.0% higher at the end of year 2 and 5.6% at the end of year 3. It is interesting to note that the coefficients were negative for the lower-intensity RCT arms (no scale and polish and 1 scale and polish), whereas they were positive for the 2 scale and polish arm.

**Table 3:** Fixed-Effects Interval Regression of Willingness to Pay

	Full Sample		No S&P		1 S&P		2 S&P	
	Coefficient	*P* Value	Coefficient	*P* Value	Coefficient	*P* Value	Coefficient	*P* Value
Year 1	−0.334	0.20	−0.197	0.66	−1.355***	<0.01	0.508	0.26
Year 2	0.0938	0.72	−0.0419	0.93	−0.651	0.15	0.935**	0.04
Year 3	0.218	0.40	−0.308	0.50	−0.0985	0.83	1.087**	0.02
Constant	19.745***	<0.01	19.765***	<0.01	20.277***	<0.01	19.100***	<0.01
Observations	3,445		1,196		1,080		1,172	
Individuals	862		299		270		293	
McFadden *R*^2^	0.0009		0.0002		0.0043		0.0034	

S&P, scale and polish.

* *P* < 0.10; **P < 0.05; ***P < 0.01. Wald test for joint significance of year 1, year 2, year 3: χ^2^(*P* value): 4.93 (0.18), 0.60 (0.90), 11.33 (0.01), 7.14 (0.07).

### Comparing WTP Function over Time

Table 4 shows the regression results of the determinants of WTP at each time point. Similar characteristics were associated with WTP across the different time points. Being exempt from dental charges and being registered with larger practices was associated with a lower WTP at each time point. Using an electric toothbrush and being resident in England was associated with higher WTP at each time point. The association between age and WTP varied across the time points, with no statistically significant association at baseline and year 3 but a significant association at year 1 and 2. The Chow test results showed that we cannot reject the null hypotheses of equal coefficients in the baseline regression model and at the 3 later time points.

**Table 4:** Determinants of Willingness to Pay at Each Time Point

	Baseline		Year 1		Year 2		Year 3	
	Coefficient	*t* Statistic	Coefficient	*t* Statistic	Coefficient	*t* Statistic	Coefficient	*t* Statistic
One S&P	0.749	0.87	−0.530	−0.62	0.100	0.11	0.971	1.11
Two S&P	−0.566	−0.67	0.257	0.31	0.545	0.60	1.145	1.34
Personalized advice^a^	−0.0943	−0.13	0.0126	0.02	1.117	1.48	0.583	0.81
Aged between 35 and 44 y	−1.193	−0.89	0.990	0.75	1.593	1.11	1.702	1.24
Aged between 45 and 54 y	0.520	0.42	2.738**	2.28	2.281*	1.74	1.471	1.18
Aged between 55 and 64 y	0.742	0.60	2.490**	2.07	2.483*	1.89	1.939	1.55
Aged 65 y and older	−0.936	−0.74	0.886	0.72	2.436*	1.81	1.728	1.36
Male	0.0961	0.13	0.482	0.66	0.296	0.37	0.608	0.80
Exempt from dental charges	−3.497***	−3.65	−3.933***	−4.04	−4.199***	−3.94	−4.736***	−4.62
Uses electric brush	2.498***	3.40	2.344***	3.28	1.679**	2.18	2.651***	3.65
Practice employs a hygienist	0.512	0.59	0.657	0.76	−0.101	−0.11	0.161	0.18
England	2.416***	2.89	1.569*	1.90	2.919***	3.24	1.865**	2.19
Constant	18.75***	10.88	16.63***	9.86	15.45***	8.37	15.92***	9.09
Observations	862		862		862		862	
McFadden *R*^2^	0.014		0.013		0.012		0.015	
Chow test (v. baseline) χ^2^ (*P* value)			6.54 (0.92)		8.78 (0.79)		8.55 (0.81)	

a This variable controls for the randomization of practices to providing routine or personalized oral hygiene advice.

* *P* < 0.10; **P < 0.05; ***P < 0.01.

### Individual-Level Changes

Table 5 shows the changes in bid amount chosen between years 1, 2, and 3 and baseline. A considerable proportion of respondents changed their bid amount. This includes about 15% who decreased their bid amount by 2 or more intervals and between 12.9% and 15.2% who increased their bid amount by 2 or more intervals. The proportion increasing and decreasing their bids were roughly similar, which explains why the mean was stable even though there were many individual changes. Table 5 also shows the difference between maximum and minimum bid amount chosen across all years. In total, 142 respondents (15.6%) chose the same bid amount throughout. About 57% of respondents changed their bid amount by 1 or 2 intervals across all years. A smaller proportion (27.3%) changed their bid amount by 3 or more intervals across all years. Appendix 5 shows the bid amounts chosen at each time point by chosen baseline bid amount. The majority of respondents are clustered on or just beside the diagonal line (shaded in gray), which represents the same bid amount chosen at both time points. However, for several other respondents, the difference between amounts chosen was considerable.

**Table 5:** Individual-Level Changes

	Year 1 v. Baseline		Year 2 v. Baseline		Year 3 v. Baseline	
Change in Bid Amount Chosen	*n*	%	*n*	%	*n*	%
No change	387	42.6	363	39.9	348	38.3
1 decrease	134	14.7	122	13.4	112	12.3
2 or more decreases	133	14.6	139	15.3	135	14.9
1 increase	138	15.2	147	16.2	183	20.1
2 or more increases	117	12.9	138	15.2	131	14.4
Interval Difference between Highest and Lowest Bid Amount Chosen across All Years	*n*	%				
0	142	15.6				
1	297	32.7				
2	222	24.4				
3	158	17.4				
4	67	7.4				
5	9	1.0				
6	12	1.3				
7	2	0.2				

### Robustness Checks

The robustness checks reestimated the fixed-effects interval regression model of WTP for different samples. The results are reported in Table 6 (full regression results are reported in Appendix 6). The results were generally similar across the different specifications. WTP was £0.45 lower at the end of year 1 compared with baseline when using the unbalanced panel.

**Table 6:** Robustness Checks

	Excluding Zeros		Unbalanced Panel	
	Coefficient	*P* Value	Coefficient	*P* Value
Year 1	−0.229	(0.36)	−0.454**	(0.03)
Year 2	0.230	(0.36)	−0.0133	(0.95)
Year 3	0.258	(0.30)	0.217	(0.31)
Constant	19.963***	(<0.01)	20.321***	(<0.01)
Observations	3365		5255	
Individuals	856		1743	
McFadden *R*^2^	0.0010		0.04382	

* *P* < 0.10; **P < 0.05; ***P < 0.01. Wald test for joint significance of year 1, year 2, year 3; χ^2^(*P* value): 5.00 (0.17); 10.60 (0.01).

## Discussion

The aim of this article was to test the stability of WTP values over time in a familiar health care good over a long time period. We found that both mean WTP and the WTP function were stable over a 3-y time period (measured at 4 time points). We also examined whether treatment allocation to different treatment intensities (0, 1, or 2 treatments per year) influenced WTP. Any effects are likely to be due to the allocation itself rather than differences in experience given that scale and polish is a familiar good and participants were part of a pragmatic trial (scale and polish was not withheld from patients requesting it). The findings suggest that preference estimates are generalizable beyond the moment when they are collected and have a reasonable shelf life. Our findings are in line with previous studies, which have typically used shorter time periods to test stability.^4–12^ We found that random allocation in an RCT to different treatment intensities did not have a consistent impact on WTP. It is interesting to note, though, that WTP was lower compared with baseline in the no scale and polish arm and the 1 scale and polish arm, which is line with the resentful demoralization hypothesis.^16,17^ However, only 1 of these effects was statistically significant.

Although mean WTP was generally stable, there were a substantial number of individual-level changes. Some of these changes may be due to imprecise preferences. However, a proportion of respondents changed their bid amount by 2 or more intervals. The proportion of respondents increasing and decreasing their bid amounts was similar, which explains why the mean WTP was stable despite a substantial number of individual-level changes.

Stable WTP values do not necessarily imply that the estimates reflect individuals’ true preferences. This requires external validity tests, which are beyond the scope of this article. It could be argued that the use of heuristics may have resulted in stable WTP estimates. Although it is likely that heuristics have been used by at least some respondents, we think it is unlikely to be the main reason for stable WTP values. First, there is unlikely to be a consistent relationship between individual characteristics and WTP if the majority of the sample used heuristics. Second, individual-level WTP values would also be expected to be stable, which was not the case in our study. We did find some possible evidence of a prominence effect and cost-based responses (WTP being higher in the region with higher user charges for dental care), suggesting that the external validity of the WTP estimates should be examined in future research.

Any unexpected changes in WTP values in health care may be due to the elicitation method itself and/or the unfamiliarity with the good. Our study tested the stability of WTP values in a familiar health care good to test whether the method itself can produce stable estimates. Individuals in our sample have experienced scale and polish (and therefore more likely to have complete preferences), and unlike other NHS services, many patients must pay a co-charge and are therefore used to considering their WTP for this service. It is important to test whether stable WTP values can be estimated for unfamiliar or less familiar health care goods.

There are several limitations to this study. First, the study was conducted using RCT participants. Individuals who consent to take part in an RCT may be atypical and more engaged and more likely to complete questionnaires in a consistent manner. Second, the WTP questions were asked as part of a relatively large self-complete questionnaire. The WTP question had to be short, and it was not possible to identify protest responses or include techniques that have been shown to improve response validity such as a cheap talk script.^33–35^ However, this may be less important when the service is familiar and most participants are used to paying. Third, the payment card CV method was used rather than the dichotomous choice method, which is the method recommended in the National Oceanic and Atmospheric Administration report.^17^ The payment card CV method and open-ended methods more generally are commonly used in health. In a recent review of the determinants of WTP for health services using the CV method, about 25% of papers used the payment card CV method and 37% used open-ended methods more generally.^36^ It is therefore important to test stability using the payment card CV method. However, results from the payment card CV method cannot necessarily be generalized to other elicitation formats, as each format has its own bases and limitations. The payment card method has been shown to have a number of biases, including range bias. It should be noted that in this study, these biases associated are likely to be constant across arms and time. Future research should examine stability in WTP for health care using other elicitation methods. Also, stability was tested using data from an RCT. It is important to examine stability in other samples, including a general population sample. Fourth, only about half of the sample had a complete set of WTP responses. The missing WTP values were mainly due to survey nonresponse rather than to item nonresponse to the CV question. Fifth, information on income was not available. Income is an important determinant of WTP, and the analysis should therefore ideally control for changes in income. However, the analysis did include a proxy for income (exemption from dental charges).

## Conclusion

We found that WTP values for scale and polish elicited using a payment card CV question were stable over time. This suggests that WTP values are transferable and can be used in cost-benefit analyses in time periods other than the one in which the WTP values were elicited. Future research should explore the stability of WTP values for other less familiar health care services, in other populations including a general population sample, and using different elicitation methods such as the dichotomous choice CV method and discrete choice experiments.

## Supplemental Material

sj-docx-1-mdm-10.1177_0272989X241249654 – Supplemental material for Stability of Willingness to Pay: Does Time and Treatment Allocation in a Randomized Controlled Trial Influence Willingness to Pay? Supplemental material, sj-docx-1-mdm-10.1177_0272989X241249654 for Stability of Willingness to Pay: Does Time and Treatment Allocation in a Randomized Controlled Trial Influence Willingness to Pay? by Marjon van der Pol, Verity Watson and Dwayne Boyers in Medical Decision Making

## Notes

i. There were 4 postrandomization exclusions in which randomization took place in error.

ii. Exemption status is taken from routine records (administrative dental claims data, linked to trial participant characteristics as part of the study).

iii. Exempt patients are those who receive income support, universal credit, or tax credit exemptions; are younger than 18 y; are receiving pension credit; or are pregnant or have given birth in the past 12 mo
