# Supplementary material for: Stability of Willingness to Pay: Does Time and Treatment Allocation in a Randomized Controlled Trial Influence Willingness to Pay?
Source: Med Decis Making. 2024 May 13;44(5):470–80. doi: 10.1177/0272989X241249654 (PMC11282685; doi:10.1177/0272989X241249654)
Supplement: sj-docx-1-mdm-10.1177_0272989X241249654 – Supplemental material for Stability of Willingness to Pay: Does Time and Treatment Allocation in a Randomized Controlled Trial Influence Willingness to Pay? [file sj-docx-1-mdm-10.1177_0272989X241249654.docx]

**Appendix 1.** Characteristics of participants and complete cases

|  | (Some) missing | | Complete WTP | |  | |  |
| --- | --- | --- | --- | --- | --- | --- | --- |
|  | N | % | N | % | χ^2^ (p-value)* | |  |
| Age | | | | | | 220.87 (0.00) | |
| <35 | 345 | 35.8 | 113 | 12.4 |  | |  |
| 35-44 | 205 | 21.3 | 133 | 14.6 |  | |  |
| 45-54 | 206 | 21.4 | 217 | 23.9 |  | |  |
| 55-64 | 99 | 10.3 | 239 | 26.3 |  | |  |
| >=65 | 108 | 11.2 | 207 | 22.8 |  | |  |
| Gender | | | | | | 0.21 (0.65) | |
| Female | 611 | 63.4 | 586 | 64.5 |  | |  |
| Male | 352 | 36.6 | 323 | 35.5 |  | |  |
| Exempt from dental charges | | | | | | 72.79 (0.00) | |
| Non-exempt | 659 | 68.4 | 774 | 85.1 |  | |  |
| Exempt | 304 | 31.6 | 135 | 14.9 |  | |  |
| Uses electric brush | 21.01 (0.00) | | | | | | |
| No | 656 | 68.0 | 584 | 64.2 |  | |  |
| Yes | 265 | 27.5 | 311 | 34.2 |  | |  |
| Missing | 43 | 4.5 | 14 | 1.5 |  | |  |
| Practice employs a hygienist | | | | | | 1.69 (0.19) | |
| No | 252 | 26.1 | 214 | 23.5 |  | |  |
| Yes | 712 | 73.9 | 695 | 76.5 |  | |  |
| Country |  |  |  |  | 1.22 (0.27) | |  |
| Scotland | 703 | 72.9 | 642 | 70.6 |  | |  |
| England | 261 | 27.1 | 267 | 29.4 |  | |  |

^*^ Pearson Chi -square test for differences between some missing and complete WTP samples.

**Appendix 2.** Frequencies of willingness to pay responses across all time points and by RCT arm

| Bid amount | **No S&P** | | **One S&P** | | **Two S&P** | | **Total** | |
| --- | --- | --- | --- | --- | --- | --- | --- | --- |
| (£) | N | % | N | % | N | % | N | % |
| **Baseline** |  |  |  |  |  |  |  |  |
| 0 | 8 | 2.8 | 6 | 2.1 | 7 | 2.3 | 21 | 2.3 |
| 1 | 1 | 0.4 | 0 | 0.0 | 0 | 0.0 | 1 | 0.1 |
| 5 | 20 | 7.0 | 22 | 7.7 | 29 | 9.6 | 71 | 7.8 |
| 10.5 | 64 | 22.5 | 45 | 15.8 | 72 | 23.8 | 181 | 19.9 |
| 15 | 79 | 27.8 | 60 | 21.1 | 63 | 20.8 | 202 | 22.2 |
| 17.5 | 20 | 7.0 | 22 | 7.7 | 13 | 4.3 | 55 | 6.1 |
| 20 | 86 | 30.3 | 94 | 33.1 | 75 | 24.8 | 255 | 28.1 |
| 30 | 36 | 12.7 | 28 | 9.9 | 38 | 12.5 | 102 | 11.2 |
| 50 | 8 | 2.8 | 5 | 1.8 | 6 | 2.0 | 19 | 2.1 |
| 75 | 0 | 0.0 | 2 | 0.7 | 0 | 0.0 | 2 | 0.2 |
| **Year 1** |  |  |  |  |  |  |  |  |
| 0 | 9 | 3.2 | 6 | 2.1 | 7 | 2.3 | 22 | 2.4 |
| 5 | 19 | 6.7 | 19 | 6.7 | 20 | 6.6 | 58 | 6.4 |
| 10.5 | 81 | 28.5 | 63 | 22.2 | 81 | 26.7 | 225 | 24.8 |
| 15 | 60 | 21.1 | 76 | 26.8 | 51 | 16.8 | 187 | 20.6 |
| 17.5 | 24 | 8.5 | 7 | 2.5 | 12 | 4.0 | 43 | 4.7 |
| 20 | 89 | 31.3 | 80 | 28.2 | 89 | 29.4 | 258 | 28.4 |
| 30 | 30 | 10.6 | 31 | 10.9 | 34 | 11.2 | 95 | 10.5 |
| 50 | 9 | 3.2 | 2 | 0.7 | 9 | 3.0 | 20 | 2.2 |
| 75 | 1 | 0.4 | 0 | 0.0 | 0 | 0.0 | 1 | 0.1 |
| **Year 2** |  |  |  |  |  |  |  |  |
| 0 | 6 | 2.1 | 8 | 2.8 | 10 | 3.3 | 24 | 2.6 |
| 1 | 0 | 0.0 | 1 | 0.4 | 0 | 0.0 | 1 | 0.1 |
| 5 | 27 | 9.5 | 12 | 4.2 | 17 | 5.6 | 56 | 6.2 |
| 10.5 | 71 | 25.0 | 55 | 19.4 | 69 | 22.8 | 195 | 21.5 |
| 15 | 70 | 24.6 | 75 | 26.4 | 59 | 19.5 | 204 | 22.4 |
| 17.5 | 17 | 6.0 | 12 | 4.2 | 15 | 5.0 | 44 | 4.8 |
| 20 | 87 | 30.6 | 79 | 27.8 | 82 | 27.1 | 248 | 27.3 |
| 30 | 34 | 12.0 | 38 | 13.4 | 40 | 13.2 | 112 | 12.3 |
| 50 | 9 | 3.2 | 3 | 1.1 | 10 | 3.3 | 22 | 2.4 |
| 75 | 1 | 0.4 | 1 | 0.4 | 1 | 0.3 | 3 | 0.3 |
| **Year 3** |  |  |  |  |  |  |  |  |
| 0 | 7 | 2.5 | 3 | 1.1 | 10 | 3.3 | 20 | 2.2 |
| 1 | 0 | 0.0 | 1 | 0.4 | 0 | 0.0 | 1 | 0.1 |
| 5 | 24 | 8.5 | 14 | 4.9 | 15 | 5.0 | 53 | 5.8 |
| 10.5 | 70 | 24.6 | 54 | 19.0 | 60 | 19.8 | 184 | 20.2 |
| 15 | 75 | 26.4 | 75 | 26.4 | 66 | 21.8 | 216 | 23.8 |
| 17.5 | 23 | 8.1 | 17 | 6.0 | 20 | 6.6 | 60 | 6.6 |
| 20 | 74 | 26.1 | 79 | 27.8 | 79 | 26.1 | 232 | 25.5 |
| 30 | 45 | 15.8 | 35 | 12.3 | 42 | 13.9 | 122 | 13.4 |
| 50 | 3 | 1.1 | 5 | 1.8 | 9 | 3.0 | 17 | 1.9 |
| 75 | 1 | 0.4 | 1 | 0.4 | 1 | 0.3 | 3 | 0.3 |
| 100 | 0 | 0.0 | 0 | 0.0 | 1 | 0.3 | 1 | 0.1 |

**Appendix 3.** Summary statistics of the willingness to pay values by year and arm (using the midpoint of the bid interval)

|  | **Baseline** | **Year1** | **Year2** | **Year3** |
| --- | --- | --- | --- | --- |
| Mean | 20.9 | 20.6 | 21.3 | 21.3 |
| Median | 16.3 | 16.3 | 16.3 | 16.3 |
| Sd | 11.4 | 11.3 | 12.0 | 11.6 |
| Minimum | 0 | 0 | 0 | 0 |
| Maximum | 75 | 75 | 75 | 75 |
| N | 909 | 909 | 909 | 909 |
| **No scale and Polish** | | | | |
| Mean | 20.9 | 20.7 | 20.9 | 20.6 |
| Median | 16.3 | 16.3 | 16.3 | 16.3 |
| Sd | 11.4 | 11.8 | 12.0 | 11.1 |
| Minimum | 0 | 0 | 0 | 0 |
| Maximum | 62.5 | 75 | 75 | 75 |
| N | 322 | 322 | 322 | 322 |
| **1 Scale and Polish** | | | | |
| Mean | 21.4 | 20.0 | 21.1 | 21.5 |
| Median | 18.8 | 16.3 | 16.3 | 16.3 |
| Sd | 11.4 | 9.9 | 11.0 | 11.1 |
| Minimum | 0 | 0 | 0 | 0 |
| Maximum | 75 | 62.5 | 75 | 75 |
| N | 284 | 284 | 284 | 284 |
| **2 Scale and Polish** | | | | |
| Mean | 20.4 | 21.1 | 21.8 | 21.9 |
| Median | 16.3 | 16.3 | 16.3 | 18.8 |
| Sd | 11.5 | 11.9 | 12.8 | 12.5 |
| Minimum | 0 | 0 | 0 | 0 |
| Maximum | 62.5 | 62.5 | 75 | 75 |
| N | 303 | 303 | 303 | 303 |

**Appendix 4.** Full interval regression results

|  | Full sample |  | No S&P |  | One S&P |  | Two S&P |  |
| --- | --- | --- | --- | --- | --- | --- | --- | --- |
|  | Coefficient | p-value | Coefficient | p-value | Coefficient | p-value | Coefficient | p-value |
| Year 1 | -0.334 | (0.20) | -0.197 | (0.66) | -1.355*** | (<0.01) | 0.508 | (0.26) |
| Year 2 | 0.0938 | (0.72) | -0.0419 | (0.93) | -0.651 | (0.15) | 0.935** | (0.04) |
| Year 3 | 0.218 | (0.40) | -0.308 | (0.50) | -0.0985 | (0.83) | 1.087** | (0.02) |
| Exempt from dental charges | -0.160 | (0.83) | -0.0524 | (0.97) | 0.204 | (0.88) | -0.300 | (0.80) |
| Uses electric brush | 0.771* | (0.08) | 0.358 | (0.63) | 0.936 | (0.24) | 1.202 | (0.12) |
| Constant | 19.745*** | (<0.01) | 19.765*** | (<0.01) | 20.277*** | (<0.01) | 19.100*** | (<0.01) |
| Observations | 3445 |  | 1196 |  | 1080 |  | 1172 |  |
| Individuals | 862 |  | 299 |  | 270 |  | 293 |  |
| McFadden R^2^ | 0.0009 |  | 0.0002 |  | 0.0043 |  | 0.0034 |  |

* p<0.10; ** p<0.05; *** p<0.01

**Appendix 5.** Chosen bid amount at year 1, 2 and 3 by baseline bid amount (N)

| Bid amount | Year 1 | | | | | | | | | |
| --- | --- | --- | --- | --- | --- | --- | --- | --- | --- | --- |
| (in £) | 0 | 1 | 5 | 10.5 | 15 | 17.5 | 20 | 30 | 50 | 75 |
| Baseline |  |  |  |  |  |  |  |  |  |  |
| 0 | 10 | 0 | 2 | 5 | 2 | 0 | 2 | 0 | 0 | 0 |
| 1 | 0 | 0 | 1 | 0 | 0 | 0 | 0 | 0 | 0 | 0 |
| 5 | 1 | 0 | 24 | 29 | 10 | 2 | 5 | 0 | 0 | 0 |
| 10.5 | 5 | 0 | 15 | 97 | 33 | 6 | 21 | 3 | 1 | 0 |
| 15 | 3 | 0 | 10 | 56 | 72 | 6 | 51 | 3 | 1 | 0 |
| 17.5 | 1 | 0 | 2 | 3 | 12 | 14 | 21 | 1 | 1 | 0 |
| 20 | 2 | 0 | 4 | 29 | 49 | 13 | 119 | 39 | 0 | 0 |
| 30 | 0 | 0 | 0 | 5 | 9 | 2 | 32 | 44 | 9 | 1 |
| 50 | 0 | 0 | 0 | 1 | 0 | 0 | 6 | 5 | 7 | 0 |
| 75 | 0 | 0 | 0 | 0 | 0 | 0 | 1 | 0 | 1 | 0 |
|  | **Year 2** | | | | | | | | | |
|  | 0 | 1 | 5 | 10.5 | 15 | 17.5 | 20 | 30 | 50 | 75 |
| Baseline |  |  |  |  |  |  |  |  |  |  |
| 0 | 9 | 0 | 1 | 5 | 4 | 0 | 1 | 1 | 0 | 0 |
| 1 | 0 | 0 | 1 | 0 | 0 | 0 | 0 | 0 | 0 | 0 |
| 5 | 4 | 1 | 22 | 27 | 8 | 0 | 6 | 2 | 1 | 0 |
| 10.5 | 6 | 0 | 16 | 83 | 44 | 5 | 20 | 7 | 0 | 0 |
| 15 | 2 | 0 | 9 | 46 | 72 | 8 | 54 | 10 | 1 | 0 |
| 17.5 | 0 | 0 | 1 | 5 | 12 | 17 | 14 | 6 | 0 | 0 |
| 20 | 3 | 0 | 5 | 24 | 53 | 10 | 114 | 42 | 4 | 0 |
| 30 | 0 | 0 | 0 | 4 | 10 | 2 | 33 | 41 | 10 | 2 |
| 50 | 0 | 0 | 1 | 1 | 1 | 2 | 5 | 3 | 5 | 1 |
| 75 | 0 | 0 | 0 | 0 | 0 | 0 | 1 | 0 | 1 | 0 |
|  | **Year 3** | | | | | | | | | |
| (in £) | 0 | 1 | 5 | 10.5 | 15 | 17.5 | 20 | 30 | 50 | 75 |
| Baseline |  |  |  |  |  |  |  |  |  |  |
| 0 | 6 | 0 | 5 | 4 | 4 | 0 | 2 | 0 | 0 | 0 |
| 1 | 0 | 0 | 1 | 0 | 0 | 0 | 0 | 0 | 0 | 0 |
| 5 | 3 | 1 | 20 | 31 | 12 | 0 | 4 | 0 | 0 | 0 |
| 10.5 | 5 | 0 | 12 | 72 | 55 | 10 | 22 | 5 | 0 | 0 |
| 15 | 2 | 0 | 8 | 40 | 75 | 19 | 43 | 13 | 2 | 0 |
| 17.5 | 0 | 0 | 1 | 9 | 9 | 15 | 20 | 1 | 0 | 0 |
| 20 | 3 | 0 | 3 | 23 | 53 | 15 | 107 | 48 | 3 | 0 |
| 30 | 1 | 0 | 2 | 4 | 8 | 1 | 28 | 49 | 8 | 1 |
| 50 | 0 | 0 | 1 | 1 | 0 | 0 | 7 | 6 | 3 | 1 |
| 75 | 0 | 0 | 0 | 0 | 0 | 0 | 0 | 0 | 1 | 1 |

**Appendix 6.** Robustness checks

|  | Excluding zero | | Unbalanced panel | |
| --- | --- | --- | --- | --- |
|  | Coefficient | p-value | Coefficient | p-value |
| Year 1 | -0.229 | (0.36) | -0.454** | (0.03) |
| Year 2 | 0.230 | (0.36) | -0.0133 | (0.95) |
| Year 3 | 0.258 | (0.30) | 0.217 | (0.31) |
| Exempt from dental charges | -0.104 | (0.88) | -0.312 | (0.56) |
| Uses electric brush | 0.706* | (0.09) | 0.676* | (0.06) |
| Constant | 19.963*** | (<0.01) | 20.321*** | (<0.01) |
| Observations | 3365 |  | 5255 |  |
| Individuals | 856 |  | 1743 |  |
| McFadden R^2^ | 0.0010 |  | 0.0438 |  |

* p<0.10; ** p<0.05; *** p<0.01;
